# Losing Legs to Losing Everything: How Neglecting Holistic Health Devastated a Lower-limb Amputee

**DOI:** 10.7759/cureus.6275

**Published:** 2019-12-02

**Authors:** Takumi Kawashita, Teevit Dunnsiri, Sara Shu, Patrick Tran, Davin Agustines

**Affiliations:** 1 Physical Medicine and Rehabilitation, Western University of Health Sciences, Irvine, USA; 2 Psychiatry, Olive View - University of California, Los Angeles Medical Center, Los Angeles, USA; 3 Internal Medicine, Western University of Health Sciences, College of Osteopathic Medicine of the Pacific, Pomona, USA; 4 Psychiatry, David Geffen School of Medicine at the University of California, Los Angeles, USA; 5 Psychiatry, Olive View - University of California Los Angeles Medical Center, Los Angeles, USA

**Keywords:** patient-centered care, depression, survey, mind, body, amputation, physical medicine and rehab, psychiatry, holistic approach, holistic care

## Abstract

Holistic care means addressing the patient as a person; providing high-quality care by focusing on individual needs. Our goal is to implement a survey that quantifies the patients’ physical, mental, and spiritual health to enable improvements in client-centered therapy in lower-limb amputees. For this, we worked with a 43-year-old Hispanic male with a medical history of insulin-dependent diabetes complicated by sequential lower limb amputations. The second amputation cost him his job and left him homeless. The patient was hospitalized after developing severe depression, to the point that he had command auditory hallucinations to kill himself. He was discharged back into the community after a three-week hospitalization. However, he was readmitted to the hospital a week later due to a resurgence of suicidal ideation. Our team engaged the patient using the “Holistic Health and Wellness Survey” of Raymond W. Smith, which we used to assess and address various domains of his mental, spiritual, and physical health. We were able to create obtainable goals for the patient for each category on which he scored low in the health and wellness survey. The patient’s overall health and attitude improved substantially through his client-centered therapy, which addressed his quantified health needs; and he began to take an active role in developing short- and long-term goals that he found attainable as he adjusted to life as a double-amputee.

This case illustrates the potential for improving client-centered therapy in lower-limb amputees. We believe that providers may benefit from implementing this health and wellness survey to better assess how to provide client-centered care for their patients.

## Introduction

Holistic care is a flourishing mission statement in modern healthcare, but it is not a new idea. The founder of osteopathic medicine, Dr. Andrew Taylor Still, believed that a consideration of all elements of a person’s body, mind, and spirit should be incorporated into their care. This type of holistic attention addresses the patient as a person and provides high-quality care by focusing on their individual needs [1].

Limited in mobility, many lower-limb amputees struggle with limited participation in society [2]. In severe cases, amputees may develop psychiatric symptoms such as depression and anxiety [3]. Despite improvements in the field of physical medicine and rehabilitation, the emotional hardships of lower-limb amputees are often overlooked [4]. Client-centered therapy helps patients to achieve change through an emphasis on their interests, emotional care, and goal-oriented problem-solving. Client-centered therapy is at the heart of holistic care: taking a mind-body approach to improving patient outcomes. At present, there are no set protocols or significant quantifying measurements to track emotional and social progress in lower-limb amputees due to the subjective nature of patients’ individual impediments. This paper discusses how implementing a survey that quantifies patients’ physical, mental, and spiritual health can further improve client-centered therapy in lower-limb amputees.

## Case presentation

Mr. H is a 43-year-old Hispanic male with a history of insulin-dependent type 2 diabetes, complicated by bilateral leg amputations and previous hospitalization for depression, with suicidal ideations and command auditory hallucinations of killing himself. He presented to the psychiatric emergency department after telling the residential staff that he was hearing voices and planned to throw himself in front of a bus.

Four months before admission, Mr. H’s diabetes had progressed significantly, and he required amputation of his right leg due to complications from his diabetes. This further debilitated him, as he had had a below-knee amputation of his left leg eight years previously, as a result of not managing his diabetes. Around this same time, he began hearing unknown female voices at night, telling him to kill himself by jumping in front of a bus. Since Mr. H had begun to hear voices, he had been thinking about committing suicide. He had never had these thoughts previously. Unable to work as a car detailer and manage his depression, Mr. H had eventually used up his savings and became homeless. The voices did not go away and his mood worsened, and he eventually came to the urgent care clinic in June asking for help. He was subsequently brought in by ambulance to the hospital and placed on 5150 hold for “danger to self.” He was held in the psychiatric emergency department until being transferred to the inpatient psychiatry unit in July for further medical stabilization.

After a few weeks of hospitalization, Mr. H was stabilized and discharged on maintenance medications of fluoxetine 60 mg daily, olanzapine 20 mg daily, and gabapentin 600 mg three times daily for major depressive disorder with psychotic features. Mr. H said that his mood had improved and the auditory hallucinations had lessened. He was accepted and discharged to an assisted living facility as part of Housing for Health, a program that provides permanent supportive housing for homeless patients.

In August, the patient reported worsening auditory hallucinations, commanding him to kill himself. Although he had a son and a girlfriend, he did not have anyone to talk to about the voices or his suicidal ideation, ultimately leaving him feeling lonely. His thoughts of suicide were prompted primarily by the voices, but when he considered acting on this, it was due to sadness about his immobility, his diabetes, the voices, and his financial stressors due to not being able to work. He did not know what to do when he had these thoughts, and he was unable to describe what had prevented him from committing suicide. He had engaged in activities for distraction, such as making the bed or listening to music, but he felt the voices were overpowering. When asked about his interests, he said that he used to enjoy drawing but he has not drawn in a while because he has not been in the mood to do so. The patient’s mental health was initially monitored with fluoxetine 60 mg daily, aripiprazole 20 mg daily, mirtazapine 30 mg nightly, and lithium 30 mg nightly.

Throughout the hospitalization, Mr. H was taken care of by the interdisciplinary team that includes the psychiatrists, nurses, physical therapists, nutritionists, the case manager, occupational therapist, and social worker. In the first 12 days after admission, Mr. H’s overall health did not improve, and the auditory hallucinations, depression, and immobility continued. He evidenced a lack of motivation to feel better, and he had no interest in talking to anyone. As Mr. H did not want to talk to us, we did not understand his needs or goals. Our team engaged Mr. H using the “Holistic Health and Wellness Survey” of Raymond W. Smith. This survey consists of three health domains: “mind,” “body,” and “spirit.” The first (mind) concerns the patient’s mental and emotional health. The second (body) focuses on the patient’s physical and environmental health. The third (spirit) is the patient’s spiritual and social health. Each domain consists of 25 questions, and the patient answers each question using a scoring system of between 0 (“strongly disagree”) and 5 (“strongly agree”). Using this survey, we assessed various domains of the patient’s mental, spiritual, and physical health. We were able to create obtainable goals for Mr. H for each category on which he scored low on the health and wellness survey. For example, Mr. H gave himself a low score for items 1 (“I maintain a healthy diet”) and 3 (“I am within 20% of my healthy, ideal body weight”) in the “body” category, both of these items describing an inability to maintain a healthy diet. In response, we advised him not to eat afternoon snacks or receive extra food from his peers. In the “mind” category, Mr. H gave himself a low score for item 13 (“I maintain peace of mind and tranquility”), indicating that he feels anxious. Mr. H rarely showered in the ward, so we advised him to shower every day because this is known to give feelings of warmth, relaxation, and refreshment [5]. Finally, in the “spirit” category, Mr. H gave himself a low score for item 24 (“I feel a sense of belonging to a group or community”), indicating that he did not engage in social interactions. We advised Mr. H to attend group activities and interact with other patients in the ward every day. Mr. H’s overall health and attitude improved substantially due to the client-centered therapy that addressed his quantified health needs, and he began to take an active role in developing short- and long-term goals that he found attainable as he adjusted to life as a double-amputee.

After this hospitalization, he hopes that he will be able to think more clearly and obtain a new prosthesis for his right leg, which would enable him to restart work as a car detailer.

## Discussion

Losing a limb has a profound impact on physical, mental, and spiritual health. The association between lower limb loss and impaired functional mobility is well-documented. Lower limb loss is also associated with other physical health problems such as phantom limb and back pain [6-7]. Limited in mobility, many lower-limb amputees are dissatisfied with limited participation in society [2]. In severe cases, amputees may develop psychiatric symptoms such as depression and anxiety [3].

Worse health outcomes are associated with poor rehabilitation results in lower-limb amputees [6]. In one study, lower-limb amputees with depression commonly reported less use of a prosthetic limb and lower self-rated overall health [8]. Another study found that an assessment of health indicators, such as physical, mental, and social function, may improve rehabilitation outcomes in lower-limb amputees [9]. Because limited mobility often requires long periods of treatment with physiatrists and physical therapists following limb amputation, healthcare professionals must understand the amputees’ overall health to ensure their optimal physical rehabilitation. Despite improvements in the field of physical medicine and rehabilitation - and a recognition of the importance of physical, mental, and social issues related to the rehabilitation of people with lower-limb amputation - the emotional hardships of lower-limb amputees are still often overlooked [4,10].

Client-centered therapy helps patients achieve change through an emphasis on patient interests, emotional care, and goal-oriented problem-solving. Client-centered therapy is at the heart of holistic care: taking a mind-body approach to improve patient outcomes. At present, there are no set protocols or significant quantifying measurements to track emotional and social progress in lower-limb amputees due to the subjective nature of patients’ individual impediments.

In the beginning, Mr. H had no interest in improving his health. His lack of motivation and unwillingness to communicate with the healthcare team made it more difficult to treat him. However, by implementing the “Holistic Health and Wellness Survey,” we were able to understand Mr. H and create obtainable goals for him in each category in which he scored low: mind, body, and spirit. Obtainable goals are tasks that almost anyone can tackle. These simple, attainable tasks can include skipping afternoon snacks, showering every day, and participating in group activities. Client-centered therapy substantially improved Mr. H’s overall health and attitude because the survey was able to address his quantified health needs. He began to take an active role in developing short- and long-term goals that he found attainable as he adjusted to life as a double-amputee.

## Conclusions

Holistic care enables us to address the patient as a person and to provide high-quality care by focusing on individual needs. Mr. H’s overall health improved by implementing the “Holistic Health and Wellness Survey” and addressing his quantified health needs. This case illustrates the potential for improving client-centered therapy in lower-limb amputees in the realm of physiatry or psychiatry.
